# Case Supposed to Be Carcinoma of the Stomach

**Published:** 1867-12

**Authors:** M. A. McClelland

**Affiliations:** Knoxville, Ill.


### CASE SUPPOSED TO BE CARCINOMA OF THE STOMACH.

By M. A. McClelland, M.D., Knoxville, Ill.

Was requested to see Mr. J. B. B., a farmer, aged 62 years, June 15th, 1867. Learned from the family that he had been under treatment the past three weeks, for a bilious attack. He had never been in a bad state of health before, except at times a little dyspeptic—a trouble which would soon pass off without treatment. At present, found him suffering from an intense pain which he referred to, and above the umbilicus. Pain had existed for some time, reaching its maximum of intensity about the middle of the day. Knees flexed upon the abdomen; decubitus on right side; pulse 100, and feeble; tongue covered with a·dense yellowish-white coat; respiration hurried; some tenderness in epigastrium, and apparently a tumor on the line separating epigastrium from umbilical region, supposed to be cancerous; eructation of flatus and ejection of mucus, with portions of substance last injested was frequent. Gave morphine, sub. nit. bismuth, and postponed a more thorough examiination till the pain was in a measure relieved. Examination two hours later revealed more clearly a tumor in the lower part of epigastric region. The tumor shaded off right and left, and apparently, on the right side, passed a little downward. Having ascertained that the bowels had not been acting for ten days past, hoped that my first impression was wrong, and that I had only a fecal tumor to deal with, and, governed by this hope, I ordered castor oil, ℥j., to be repeated in four hours, and again in six hours if necessary. Morphine was only to be given during the night if the pain became insupportable. At 4 o'clock A.M., found patient had taken both oil and morphine, and was now comparatively easy. Abdominal muscles relaxed to a considerable extent. On examination, I was able to follow outline of ascending and transverse colon very distinctly, distended with fecal matter. Diagnosis then supposed to be clearly made out, and ordered cathartics to be pressed ℥j.–℥iss., castor oil every four hours. At 11 o'clock A.M., exhibited two drops croton oil, and at three-quarters past 11, gave an enema. ℞. Water Oj., lard, molasses, and salt āā, a tablespoonful. Retained ten minutes, and brought away with it some thirty scybalæ. Patient greatly relieved. Ordered castor oil, ℥j., to be given at 3 P.M., followed in two hours by another enema, which was retained about five minutes, and was discharged with about two quarts of feculent matter, which had a good, healthy odor. Patient greatly relieved, and expressed a desire for food, which was given. Outline of ascending and transverse colon lost; tumor in epigastrium indefinite; pulse fell to 80; tongue began to clean from the edges and tip, being moist from the first examination. Ordered anodynes at long intervals. Elixir of bark and iron, with lime-water and milk, as the stomach was somewhat irritable. Tumor, as before stated, indefinite, but, by deep palpation, could be made out in shape similar to a longitudinal section of a hen's egg. The evacuation of so large a quantity of fecal matter, with the relief it afforded patient, seemed to confirm the diagnosis of a fecal tumor. Being of a sanguine temperament, I begged the friends to believe I had taken a wrong view of the case when I first feared it was cancer.

Left an appointment to see the patient again on the 18th. When I returned, I found he was again laboring under considerable pain, with tumor in epigastrium more distinct. Believing it to be occasioned, to a great extent, by a reaccumulation in transverse colon, I labored during the day with oil and injections, to unload the bowels. Not having accomplished much by evening, I began to revert to my first opinion, inasmuch as he had been vomiting some grumous, slate-colored matter. Had concluded to put patient on anodynes, tonics, and lime-water, and milk diet, with the intention of again acting on the bowels, after a few days, if the case seemed to demand it. At the suggestion of a medical gentleman, who called at the time, and who, at my request, made an examination of the case, I added to the anodynes, gr. $\frac{1}{8}$, hydrarg. chloridi mite, which was ordered to be given every four hours.

On my return, the 20th, found the patient much improved. The bowels had acted slightly. Tumor still indefinitely made out in epigastrium, and referred it to contraction of recti muscles, as suggested by the medical gentleman who examined the case at my last visit. Directed anodynes, without the alterative, tonics, and nutrition continued.

Patient doing so well on the 24th, that we were only to visit him again, if summoned. The decubitus of patient was still on right side; knees not so constantly flexed upon the abdomen; tongue cleaning from tip and edges nicely; pulse 80, and full; pain trivial, yet referred to same locality; tumor as indistinct as ever. Directed a continuance of treatment.

Was summoned to see patient July 2d, and found him laboring under an aggravation of symptoms; pain severe; pulse 108, and feeble; knees and thighs strongly flexed on the abdomen; eructation of flatus; vomiting of grumous matter; semi-delirious; tumor, either from increase of volume, or emaciation of parietes of abdomen, quite distinct—so palpable, that it was easily made out by a number of gentlemen who severally called during the day. Ordered anodynes, and informed friends that early dissolution was to be expected. Patient died next day. No *post mortem* admitted.

There are several points of interest in the foregoing case. *First*, The great difficulty in making out a differential diagnosis in tumors of the abdominal cavity, especially by one who has but limited opportunities of verifying his diagnosis by a *post mortem* examination. *Second*, The strong presumptive evidence of carcinoma elicited by a tumor in the epigastric region, especially if that tumor is of a very firm, immovable character. *Third*, The rapid development of the disease, manifesting itself during the period of latency by only slight dyspeptic symptoms.

Nothing has yet been said in regard to the peculiar cachexia that is usually observed in carcinoma. In a person of that age, following an out-door occupation, the cachexia is greatly modified by such exposure, and was, in the present case, sufficient to hide the peculiar waxy hue that attends malignant disease of the human system.

